# Supplementary material for: The Evolution and Characterization of the RNA Interference Pathways in Lophotrochozoa
Source: Genome Biol Evol. 2024 May 7;16(5):evae098. doi: 10.1093/gbe/evae098 (PMC11114477; doi:10.1093/gbe/evae098)
Supplement: evae098_Supplementary_Data [file evae098_supplementary_data.zip › Supplementary_materials.zip!/selected_miRNAs/CrGi_selected_miRNAs/CrGi_NC_047560.1_3071.pdf]

Provisional ID	:	NC_047560.1_3071
Score total	:	407.5
Score for star read(s)	:	3.9
Score for read counts	:	399.8
Score for mfe	:	2.8
Score for randfold	:	1.6
Score for cons. seed	:	-0.6
Total read count	:	796
Mature read count	:	747
Loop read count	:	0
Star read count	:	49

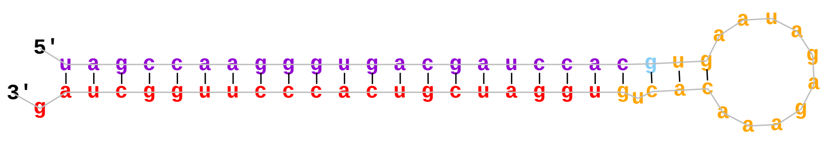

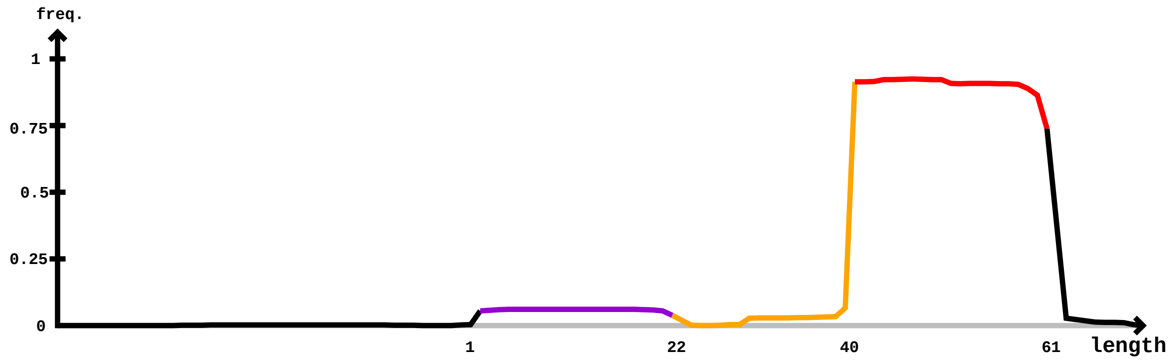


```
                                           Star                                   Mature
5'- augacuguaguauucugcugagcuacagaacuacaucuuugcuagccaagggugacgauccacgugaauagagaaacacuguggaucgucacccuuggcuagcgaagauga   -3'   obs
    augacuguaguauucugcugagcuacagaacuacaucuuugcuagccaagggugacgauccacgugaauagagaaacacuguggaucgucacccuuggcuagcgaagauga         exp
    ....((((((..((....)))))))).....((((((((((((((((((((((((((((((((((..........))).))))))))))))))))))))))))))))))).   reads     mm     sample
    ...........auucugcugagcuacagaacuaUau...........................................................................   1         1      Mt1
    .........................................cuagccaagggugacgaucUa.................................................   1         1      Mt1
    ..........................................uagccaagggugacgaucc..................................................   2         0      Mt1
    ..........................................uagccaagggugacgaucca.................................................   3         0      Mt1
    ..........................................uagccaagggugacgauccac................................................   4         0      Mt1
    ...........................................agccaagggugacgauccacg...............................................   1         0      Mt1
    .............................................ccaagggugacgauccac................................................   1         0      Mt1
    ......................................................................gagaaacacuguggaucguc.....................   2         0      Mt1
    ......................................................................gagaaacacuguggaucguca....................   2         0      Mt1
    ......................................................................gagaGacacuguggaucguca....................   1         1      Mt1
    ..............................................................................cuguggaucgucacccuCggc............   1         1      Mt1
    ................................................................................guggaucgucacccuugg.............   1         0      Mt1
    ................................................................................guggaucAucacccuuggcu...........   1         1      Mt1
    ................................................................................guggaucgucacccuuggcu...........   1         0      Mt1
    ................................................................................guggaucgucacccuuggcua..........   3         0      Mt1
    ................................................................................guggaucAucacccuuggcuag.........   1         1      Mt1
    .................................................................................uggaucgucacccuuggc............   1         0      Mt1
    .................................................................................uggaucgucacccuuggcu...........   1         0      Mt1
    .................................................................................uggaucgucacccuuggcua..........   30        0      Mt1
    .................................................................................uggaucgucacccuuggGua..........   1         1      Mt1
    .................................................................................uggaucgucacccuuggcuag.........   176       0      Mt1
    .................................................................................uggaucgucacccuuggcuGg.........   4         1      Mt1
    .................................................................................uggaucUucacccuuggcuag.........   1         1      Mt1
    .................................................................................uggaucgucacccuuggcuagc........   4         0      Mt1
    ....................................................................................aucgucacccuuggcuagcg.......   2         0      Mt1
    ..........................................................................................accAuuggcuagcgaagaug.   1         1      Mt1
    .............................................................................................cuuggcuagcgaagauga   1         0      Mt1

    ..........................................uagccaagggugacgaucca.................................................   5         0      Mt2
    ..........................................uagccaagggCgacgauccac................................................   1         1      Mt2
    ..........................................uagccaagggugacgauccac................................................   7         0      Mt2
    ......................................................................gagaaacacuguggaucguc.....................   1         0      Mt2
    ......................................................................gagaaacacuguggaucguca....................   2         0      Mt2
```

Star **Mature**

```
augacuguaguauucugcugagcuacagaacuacaucuuugcuagccaagggugacgauccacgugaauagagaaacacuguggaucgucacccuuggcuagcgaagauga
......................................................................gaCaaacacuguggaucguca....................   1    1    Mt2
.............................................................................acuguggaucgucacccuCgg.............   1    1    Mt2
...............................................................................Aguggaucgucacccuuggcu...........   1    1    Mt2
................................................................................guggaucgucacccuuggc............   1    0    Mt2
................................................................................guggaucAucacccuuggcu...........   1    1    Mt2
................................................................................guggaucgucacccuuggcua..........   2    0    Mt2
................................................................................guggaucAucacccuuggcuag.........   1    1    Mt2
.................................................................................uggaucgucacccuuggc............   5    0    Mt2
.................................................................................uggaucgucacccuuggcu...........   6    0    Mt2
.................................................................................uggaucgucacccuuggcua..........   23   0    Mt2
.................................................................................uggCucgucacccuuggcuag.........   2    1    Mt2
.................................................................................uggaucgucacccuuggUuag.........   1    1    Mt2
.................................................................................uAgaucgucacccuuggcuag.........   1    1    Mt2
.................................................................................uggaucgucacccuuggcuag.........   181  0    Mt2
.................................................................................uggaucgucacccuuggcuGg.........   2    1    Mt2
....................................................................................aucgucacccuuggcuagcg.......   1    0    Mt2
....................................................................................aucgucacccuuggcuagcga......   1    0    Mt2
..........................................................................................acccuuggcuagcgaagUu..   1    1    Mt2
..........................................................................................acccuuggcuagcgaagaug.   1    0    Mt2
...........................................................................................cccuuggcuagcgaagauga   1    0    Mt2

..............cugcugagcuacGgaacua..............................................................................   1    1    Ov2
........................................gcuagccaagggugacgauc...................................................   1    0    Ov2
..........................................uagccaGgggugacgaucc..................................................   1    1    Ov2
..........................................uagccaagggugacgaucca.................................................   2    0    Ov2
..........................................uagccaagggugacgauccac................................................   9    0    Ov2
...........................................agccaagggugacgauccaU................................................   1    1    Ov2
...................................................................auagagaGacacuguggaucguca....................   1    1    Ov2
....................................................................uagaCaaacacuguggaucgu......................   1    1    Ov2
......................................................................gagaGacacuguggaucguc.....................   1    1    Ov2
......................................................................gagaGacacuguggaucguca....................   2    1    Ov2
......................................................................gagaaacacuguggaucguca....................   3    0    Ov2
......................................................................gagaaacacuguggauUguca....................   1    1    Ov2
.......................................................................agaGacacuguggaucguca....................   1    1    Ov2
...........................................................................acacuguggaucgucacccuC...............   1    1    Ov2
................................................................................guggaucgucacccuuggcua..........   1    0    Ov2
................................................................................guggaucAucacccuuggcuag.........   1    1    Ov2
................................................................................guggaucAucacccuuggcuagcga......   1    1    Ov2
................................................................................guggaucgucacccuuggcuagcgaa.....   1    0    Ov2
.................................................................................uggaucgucacccuuggc............   2    0    Ov2
.................................................................................uggaucgucacccuuggcu...........   4    0    Ov2
.................................................................................uggaucgucacccuuggcua..........   17   0    Ov2
.................................................................................uggaucgucGcccuuggcuag.........   1    1    Ov2
.................................................................................uggaucgucaccAuggcuag.........   1    1    Ov2
.................................................................................Aggaucgucacccuuggcuag.........   1    1    Ov2
.................................................................................uggaucgucacccuuggcuag.........   105  0    Ov2
.................................................................................uggaucguUacccuuggcuag.........   1    1    Ov2
.................................................................................uggaucgucacccuuggcuGg.........   3    1    Ov2
.................................................................................uggaucgucacccuuggcuagc........   1    0    Ov2
.................................................................................Gggaucgucacccuuggcuagc........   1    1    Ov2
.................................................................................uggaucgucacccuuggcuagcU.......   1    1    Ov2
...................................................................................gaucgucacccuuggcuagc........   1    0    Ov2
....................................................................................aucgucacccuuggcuagcgaa.....   1    0    Ov2
......................................................................................cgucacccAuggcuagcgaag....   1    1    Ov2
.......................................................................................gucacccuCggcuagcgaa.....   1    1    Ov2
...........................................................................................cccuuggcCagcgaagaug.   2    1    Ov2
...........................................................................................Uccuuggcuagcgaagaug.   1    1    Ov2
...........................................................................................cccuuggcuagcgaagaCga   1    1    Ov2

........................................gcuagccaagggugacgau....................................................   1    0    Ov1
..........................................uagccaagggugacgaucca.................................................   3    0    Ov1
..........................................uagccaagggugacgauccac................................................   6    0    Ov1
............................................gccaagggugacgauccac................................................   1    0    Ov1
............................................gccaagggugacgauccacgC..............................................   1    1    Ov1
....................................................................uagaCaaacacuguggaucg.......................   1    1    Ov1
......................................................................gagaGacacuguggaucguca....................   1    1    Ov1
......................................................................gagaaacacuguggaucguca....................   2    0    Ov1
......................................................................gagaaacacuguggaucgucac...................   1    0    Ov1
................................................................................guggaucgucacccuuggcu...........   2    0    Ov1
................................................................................guggaucgucacccuuggcua..........   5    0    Ov1
```

Star   Mature

```
augacuguaguauucugcugagcuacagaacuacaucuuugcuagccaagggugacgauccacgugaauagagaaacacuguggaucgucacccuuggcuagcgaagauga
................................................................................guggaucgucacccuuggcuag.........    2    0    Ov1
................................................................................guggaucAucacccuuggcuagcga......    2    1    Ov1
.................................................................................uggaucgucacccuuggc............    3    0    Ov1
.................................................................................uggaucgucacccuuggcu...........    4    0    Ov1
.................................................................................uggaucgucacccuuggcua..........    23   0    Ov1
.................................................................................Aggaucgucacccuuggcuag.........    1    1    Ov1
.................................................................................uggaucgucacccuuggcuGg.........    2    1    Ov1
.................................................................................uggaucgucacccuuggcuag.........    99   0    Ov1
.................................................................................Cggaucgucacccuuggcuag.........    1    1    Ov1
....................................................................................aucgucacccuuggcuagcgaa.....    1    0    Ov1
..........................................................................................acccuuggcuagcgaagaug.    1    0    Ov1
```
